# Dispensing practices for psychotropic medications amongst pharmacists in Karachi, Pakistan

**DOI:** 10.1016/j.heliyon.2022.e11298

**Published:** 2022-10-28

**Authors:** Shahzain Hasan, Mujeeb U. Shad

**Affiliations:** a Neuro Mind Hospital, Sir Syed University, Sardar Ali Sabri Road, Block-5, Gulshan-e-Iqbal, Karachi, Sindh, 75300, Pakistan; b Univerity of Nevada, Las Vegas, NV, USA; c Touro University Nevada College of Osteopathic Medicine, Las Vegas, NV, USA; d Department of Psychiatry, Valley Health System, Las Vegas, NV, USA

**Keywords:** Psychiatry, Mental health, Pharmacists, Antidepressants, Dispensing

## Abstract

**Background & Objective:**

Uninformed use of medications can be dangerous especially those medications that require some level of monitoring to ensure safety and tolerability and prevent misuse, such as benzodiazepines and other psychotropic medications. In most developed countries, medications (except over-the-counter medications) are not dispensed without a physician's script. This may not be true for developing countries, like Pakistan, where nearly all medications are dispensed without a script. However, the extent and nature of script-less dispensing has never been studied. This study was designed to investigate the extent and prevalence of dispensing psychotropic medications without physicians' prescriptions, and the pharmacy practices, including the staff qualifications, to not only dispense but also recommend psychotropic medications, in Karachi, Pakistan.

**Method:**

A Cross-sectional study was conducted in Karachi over three months (September 2021 to November 2021) with 200 pharmacists in various pharmacies, using a structured questionnaire in a convenient study sample. Pharmacists from registered pharmacies were included in the study. Statistical analysis was done using the Chi-Square test of association.

**Results:**

Out of 200 pharmacists working at various locations, 89.0% did not required prescriptions to dispense medications, with benzodiazepines being the most frequently dispensed medication. Surprisingly, only 9.0% had a bachelor's in pharmacy and were qualified enough to legally dispense medications. 76.0% admitted to recommending medications to the patients. Since many of the pharmacists were not qualified enough to dispense medications, 78.5% mentioned that they did not had awareness regarding the abuse potential of psychotropic medications.

**Conclusion:**

Dispensing of psychotropic medications without prescriptions and recommending such medications has been a significant issue in the past. Our study reveals this practice to be prevalent in this part of the world, posing a serious threat to the patients. Steps should be taken by the government to ensure proper dispensing of these medications having an abuse potential to prevent harm.

## Highlights

• Dispensing of Psychotropic medications without a prescription by retail pharmacies is a common practice in Karachi, Pakistan.
• Benzodiazepines are by far the most common dispensed and recommended psychotropic medications, with majority of dispensers not being aware of the abuse potential of these control medications
• Importance of qualification, knowledge, and training of pharmacy employees are not highlighted in Pakistan and effective implementation of policies is needed.

## 1 Introduction

Mental health problems have increased remarkably in Pakistan, [1, 2] and their societal burden is worsened by non-detection, misdiagnosis, inappropriate treatment, and self-prescription. [3, 4] Unfortunately, access to qualified medical practitioners, especially those with any formal training in psychiatry, is limited in most rural and urban areas, which results in pharmacies being the first point of contact for many patients seeking health care [5]. A limited number of qualified pharmacists further increases the practice of inappropriate dispensing and recommendation of psychotropic medications.

Prescription of medications is strongly regulated in most developed countries worldwide, and only qualified pharmacy staff dispenses medications prescribed by licensed practitioners. Although an ordinance called, The Allopathic System (Prevention of Misuse) Rule [1962] clearly defines who can prescribe and dispense medications in Pakistan, non-regulated drug distribution methods are still the rule rather than expcetion [6].

In general, the primary task of a pharmacist is to provide dispensing information and counseling about the prescribed medication(s) to the patients. [7] However, these rules are not followed in developing countries as the application of pharmaceutical care is affected by time constraints, less access to medical records, poor communication among healthcare providers, insufficient number of qualified pharmacists [8], and dispensing of medications without prescriptions.

A study conducted in Pakistan outlined that the overall qualifications and knowledge of the staff working at the pharmacies were inadequate and primarily operated by the nonqualified personnel [9]; this was also seen in other developing countries of the world [10, 11, 12].

More than half of all medications sold in Pakistan are dispensed without prescriptions, [13] analgesics, antibiotics, and even benzodiazepines, and antidepressants are readily available without prescription from community pharmacies in Pakistan [14, 15, 16, 17, 18].

Script-less availability of benzodiazepines alone has been and remains a major cause of dependency and suicide in the past, often termed as 'stress relievers. [19] One of the most addictive benzodiazepines, alprazolam, was reported as the most common dispensed psychotropic medication in Pakistan Among benzodiazepines. [20] Other groups of psychotropic medications, such as antidepressants and stimulants, are also consumed significantly due to their easy availability resulting in serious health risks in the 'patients' population who tend to self-medicate themselves for various reasons/medical conditions [21].

Although a previous study in Pakistan has reported high rates of self-medication with analgesics, antipyretics, and antibiotics, it did not assess the script-less use of psychotropic medications. [22] This, to our knowledge, is the first study to investigate patterns, nature, and prevalence of script-less dispensing and treatment recommendations for psychotropic medications by the local pharmacies in Karachi, along with the qualifications of their pharmacy staff.

## 2 Methods

### 2.1 Study design and sampling

A cross-sectional study was carried out using a nonprobability convenient sampling technique from various pharmacies in the densely populated Karachi, Pakistan, from Sept 2021 till Nov 2021.

### 2.2 Criteria

#### 2.2.1 Inclusion criteria

1. Randomly selected staff members at registered pharmacies.
2. Randomly selected staff members working at hospitals, clinics, and retail pharmacies.

#### 2.2.2 Exclusion criteria

1. Pharmacy staff who refused to answer all the questions in the questionnaire.
2. Pharmacy staff who refused to consent for the study.

### 2.3 Data collection and questionnaire

A total sample of 200 was collected from employed personnel working at various pharmacies, and a structured questionnaire was used to get information on the knowledge and prescribing trends of the staff working at these pharmacies. One staff member from every pharmacy was randomly requested to fill out the questionnaire. Responses of the questionnaire were finalized after a pilot study done on fifty (50) random pharmacy staff available and comprised of questions related to their demographics, education level, frequency of dispensing and recommending psychotropic medications, knowledge of abuse potential, and requirement of prescriptions prior to dispensing these control medications. Although the primary language used to construct the questionnaire was English, it was translated into the local language (Urdu) for a better understanding of the questions for the participants. Cronbach's alpha to check the reliability of the questionnaire was 0.779. Informed consent was obtained from all the participants to include their responses in the study while maintaining anonymity.

### 2.4 Ethical consideration

Ethical approval was obtained from the review committee of District Health Officer (DHO) Karachi (South).

An informed consent form was attached with the questionnaire, which informed all the potential participants about the purpose of data collection and incorporating these findings in the study while maintaining anonymity. The questionnaire was anonymous and guaranteed the confidentiality of the study participants as it did not document any information that could link individuals to the study.

### 2.5 Statistical analysis

Data entry and statistical analysis were done using Statistical Package for Social Sciences 25.0 (SPSS v25.0). Percentages of responses were calculated for relevant variables. A chi-squared test of association was used. The significance level was *p* < 0.05.

## 3 Results

A total of 200 staff members from various pharmacies, with an average age of 36 years (SD ± 7.448), were requested to fill out a questionnaire in the various pharmacies located at hospitals, clinics, and individual retail pharmacies. As evident from Table 1, 99.5% of the subject sample were males, with 89.0% working at independent retail pharmacies. Surprisingly, when asked about education level, 72.0% of the staff responded as having a bachelor's degree other than pharmacy (e.g., BA), with only 9.0% having a degree in pharmacy (qualified pharmacists). Regardless of their qualification, almost all staff members mentioned benzodiazepines as the most dispensed psychotropic medication (99.5%) with or without prescription. Apart from just dispensing, 76.0% stated that they sometimes do recommend medications to customers with benzodiazepines again being the most common recommended psychotropic medication. With only 7.5% pharmacy staff reluctant to dispense medications without a script, 89.0% were comfortable with dispensing these medications without a prescription.

**Table 1:** Descriptive analysis and frequencies of variables in the study.

Frequencies			
Variables		Total (n = 200)	Percentage (%)
**Gender**	Male	199	99.5
	Female	1	0.5
**Location of Pharmacy**	Independent Retail Pharmacy	178	89.0
	Clinic Pharmacy	8	4.0
	Hospital Pharmacy	14	7.0
**Higher Education**	Bachelors other than Pharmacy	144	72.0
	Other degree in Pharmacy (e.g., Diploma)	23	11.5
	Doctor of Pharmacy	18	9.0
	None	15	7.5
**Most Common Dispensed Psychotropic medications**	Benzodiazepine	199	99.5
	Antidepressants	1	0.5
**Frequency of recommending medications**	Rarely (Less than 3 times in a week)	152	76.0
	Never	48	24.0
	Always	0	0
**Most Common Recommended Psychotropic Medications**	Benzodiazepine	156	78.0
	Antidepressants	0	0
	None	44	22.0
**Is Prescription required for dispensing medications?**	No Prescription Needed	178	89.0
	Sometimes	7	3.5
	Always	15	7.5
**Knowledge for Conditions for which the drug was prescribed**	No Knowledge	167	83.5
	Know a little about the disorder and the commonly prescribed medications	33	16.5
**Awareness regarding abuse potential of psychotropic medications**	No	157	78.5
	Yes	43	21.5

### 3.1 Requirement of prescriptions for psychotropic medications at different pharmacy locations

#### 3.1.1 Independent retail pharmacies

As outlined in Table 2, out of the total sample size, 178 samples were from independent retail pharmacies, which are present in a large number throughout the city and outnumber the pharmacies located in hospitals and clinics combined. Out of these 178 samples, 96.1% (n = 171) were comfortable with dispensing psychotropic medications without a prescription, while 1.1% (n = 2) required a prescription at every patient encounter to dispense medications. Some of the staff members (2.8%, n = 5) were reluctant at times to dispense medications without a prescription; however, they did admit it to let it sometimes go (*p < 0.001).*

**Table 2:** Requirement of prescriptions according to different pharmacy locations.

Requirement of Prescriptions at Different Pharmacy Locations			Is Prescription Required for Dispensing Medications?				
			No Prescription needed	Sometimes	Always	Total	p-value
**Location Of Pharmacy Setting**	**Independent Retail Pharmacy**	**Count**	171	5	2	178	<0.001
		**% Within Location of Pharmacy**	96.1%	2.8%	1.1%	100.0%	
	**Clinic Pharmacy**	**Count**	3	1	4	8	
		**% Within Location of Pharmacy**	37.5%	12.5%	50.0%	100.0%	
	**Hospital Pharmacy**	**Count**	4	1	9	14	
		**% Within Location of Pharmacy**	28.6%	7.1%	64.3%	100.0%	

#### 3.1.2 Clinic Pharmacy

Eight (8) pharmacy staff members out of the total sample size were from eight private clinics, 50% (n = 4) of which required prescription to dispense psychotropic medications; however, 37.5% (n = 3) did not require a prescription. 12.5% (n = 1) of the pharmacy staff admitted to asking for a prescription occasionally (*p < 0.001).*

#### 3.1.3 Hospital Pharmacy

A total of 14 pharmacy staff included in the study were working at a hospital, 64.3% of whom (n = 9) always required a prescription from a licensed physician to dispense psychotropic medications. 28.6% (n = 4) dispensed psychotropic medications without the script (*p < 0.001).*

### 3.2 Requirement of prescription for psychotropic medications by pharmacists according to their educational level

#### 3.2.1 Degree other than pharmacy

Surprisingly, 144 participants had qualifications other than pharmacy. Out of which, 96.5% (n = 139) dispensed psychotropic medications without prescription, while 2.1% (n = 3) always required prescriptions. 1.4% (n = 2) occasionally required prescription as evident in Table 3 *(p < 0.001*).

**Table 3:** Requirement of prescriptions by pharmacists according to their educational level.

Requirement of prescriptions according to Educational Level of Pharmacists			Is Prescription Required for Dispensing Medications?				
			No Prescription needed	Sometimes	Always	Total	p-value
**Education**	**Degree other than pharmacy**	**Count**	139	2	3	144	<0.001
		**% Within Educational Level**	96.5%	1.4%	2.1%	100.0%	
	**Other degree in Pharmacy (e.g., Diploma)**	**Count**	20	1	2	23	
		**% Within Educational Level**	87.0%	4.3%	8.7%	100.0%	
	**Doctor of Pharmacy**	**Count**	5	3	10	18	
		**% Within Educational Level**	27.8%	16.7%	55.6%	100.0%	
	**None**	**Count**	14	1	0	15	
		**% Within Educational Level**	93.3%	6.7%	0.0%	100.0%	

#### 3.2.2 Other degrees in pharmacy (e.g., diploma)

Out of the total participants, 23 had other qualifications in pharmacy (other than bachelors, e.g., Diploma). 87.0% (n = 20) of them required no prescription while 8.7% (n = 2) required a script to dispense psychotropic medications *(p < 0.001*).

#### 3.2.3 Doctor of Pharmacy

Only 18 out of the total pharmacy staff members included in the study were qualified pharmacists with a bachelor's degree. 55.6% (n = 10) of them always dispensed psychotropic medications with prescriptions, while 27.8% (n = 5) required no prescription. 16.7% (n = 3) occasionally required prescriptions *(p < 0.001).*

#### 3.2.4 No higher education

On the contrary, 15 of the participants did not have any higher qualifications, with 93.3% (n = 14) of these staff members dispensing psychotropic medications without prescriptions, while the remaining 6.7% (n = 1) occasionally required prescriptions *(p < 0.001).*

### 3.3 Most common dispensed psychotropic medication according to the location of pharmacy

#### 3.3.1 Independent retail pharmacy

As seen in Table 4, the most common dispensed psychotropic agent at all independent retail pharmacies (n = 178) was benzodiazepines, regardless of whether a prescription was required or not *(p < 0.001*).

**Table 4:** Most common dispensed psychotropic medication according to location of pharmacy.

			Most Common Dispensed Psychotropic Medication				
			Benzodiazepines	Antidepressants	Antipsychotics	Total	p- Value
**Location of pharmacy**	**Independent Retail Pharmacy**	**Count**	178	0	0	178	<0.001
		**% Within location of pharmacy setting**	100.0%	0.0%	0.0%	100%	
	**Clinic Pharmacy**	**Count**	7	1	0	8	
		**% Within location of pharmacy setting**	87.5%	12.5%	0.0%	100%	
	**Hospital Pharmacy**	**Count**	14	0	0	14	
		**% Within location of pharmacy setting**	100.0%	0.0%	0.0%	100.0%	

#### 3.3.2 Clinical pharmacy

Out of pharmacy staff working at clinic pharmacies, 87.5% (n = 7) mentioned benzodiazepine again as the most dispensed psychotropic medication, with 12.5% (n = 1) claiming antidepressants to be the most dispensed psychotropic medication at their clinic (p < 0.001).

#### 3.3.3 Hospital pharmacy

Benzodiazepine was the most common psychotropic medication dispensed at all the hospital pharmacies (n = 14) (p < 0.001).

### 3.4 Most common recommended psychotropic medication according to pharmacy location

#### 3.4.1 Independent retail pharmacy

Out of a total of 178 staff members working at retail pharmacies, Outlined in Table 5, 85.4% (n = 152) recommended benzodiazepines at some point, while 14.6% (n = 26) chose not to recommend medications to the patients. Benzodiazepine was the only recommended medication (n < 0.001).

**Table 5:** Most common recommended psychotropic medication according to location of pharmacy.

			Most Common Recommended Psychotropic Medication					
			Benzodiazepine/Anxiolytics	Antidepressant	Antipsychotics	None	Total	p- Value
**Location of Pharmacy Setting**	**Independent Retail Pharmacy**	Count	152	0	0	26	178	<0.001
		% Within Location of pharmacy	85.4%	0.0%	0.0%	14.6%	100%	
	**Clinic Pharmacy**	Count	0	0	0	8	8	
		% Within Location of pharmacy	0.0%	0.0%	0.0%	100.0%	100%	
	**Hospital Pharmacy**	Count	4	0.0%	0	10	15	
		% Within Location of pharmacy	28.6%	0.0%	0.0%	71.4%	100.0%	

#### 3.4.2 Clinic pharmacy

All clinic dispensers denied recommending any psychotropic medications to the patients (n < 0.001).

#### 3.4.3 Hospital pharmacy

Out of 15 hospital pharmacies, 71.4% (n = 10) never recommended any psychotropic medication; however, 28.6% (n = 4) recommended benzodiazepines to the patients (n < 0.001).

## 4 Discussion

The findings from this study underscore the high prevalence of script less dispensing of psychotropic medications by unsupervised and unqualified staff in Karachi, Pakistan. Only a minority (11%) of pharmacies required a script to dispense psychotropic medications, with most pharmacy staff (78.5%) were not aware of these drugs' abuse potential. Script less dispensing was even higher in retail pharmacies, and only four percent required script to prescribe psychotropic medications. However, our study found a significantly lower prevalence of script less dispensing of psychotropic medications in four hospital pharmacies included in this study (89% versus 29%).

More alarming was the recommendation of psychotropic medications by the unqualified retail pharmacy staff (85%). In addition, only 9.0% (n = 18) out of 200 participants surveyed were trained pharmacist with a bachelor's degree in pharmacy. At the same time, the rest were not qualified enough to dispense medications, which significantly compromises patients' safety. Several prior studies have reported such practices in other developing countries. [11, 23, 24] The study showed an inverse relationship between the educational level of the pharmacy staff and the risk for dispensing psychotropic medications without a script.

Uninformed and unsupervised use of psychotropic medications carries a risk for potentially lethal adverse effects and abuse. A report published by United Nations on Drug use in Pakistan (2013) outlined that 1.5 million people had misused sedatives and tranquilizers in 2012 for non-therapeutic purposes, with the majority consuming these medications more than once weekly, while a smaller number used them every day. [25] These findings were similar to those from the national assessment of drug use in Pakistan in 2006, which reported that sedatives, such as benzodiazepines, were amongst the most abused substances. [26] Similar study conducted in Saudi Arabia showed alarming rates of dispensing of controlled medications that are easily available and poses a threat to the population [27].

Unqualified pharmacy staff also lacks knowledge of medication-related adverse effects, the risk for withdrawal and tolerance, effective dosages, therapeutic drug monitoring, or drug interactions. [28] Additionally, none of the pharmacies kept any record of the date and quantity of dispensed medications, thus significantly elevating the risk for addiction and potentially lethal overdose. The easy availability of benzodiazepines explains the frequent reports of benzodiazepine overdosing and self-poisoning cases in Pakistan [19].

Although over the counter (OTC) medications that don't require a script or a physician's expertise to manage minor ailments can reduce the healthcare burden in any country, [29] the script less availability of complex medications, such as benzodiazepines, stimulants, and antidepressants, carries a significant risk for untoward outcomes in our patients, including suicide and overdosing [19].

A developing country, such as Pakistan, needs to develop preventive programs and educate chemists/dispensers/pharmacy staff to minimize harm to the general population by carefully dispensing psychotropic medications alongside reshaping and implementing drug regulation policies and dispensing practices. In addition, the current stressful times require public education on mental healthcare and resources to elevate the level of psychiatric care, reduce harmful adverse effects, and increase the quality of life and productivity.

## 5 Limitations

The results from this study are based on a cross-sectional survey using a questionnaire developed for this study and should be interpreted with caution. Other limitations are as follows:

1. The data were collected by using a nonprobability convenient sampling technique, which may lead to selection bias.
2. Pharmacy staff members filled out responses in the questionnaire based on their memory, which is subject to recall bias.
3. Staff working at various pharmacies may have reported a smaller number of dispensed medications without prescriptions or a smaller number of recommended medications to be under-reported.
4. The number of retail pharmacies is more than hospital or clinic pharmacies; hence, most responses were from independent retail ones and cannot be projected since the number of different pharmacies at various places may vary.
5. Due to lack of finances and time, we restricted our sample size to 200.
6. It is possible that many pharmacy personnel did not fully disclose prescribing or dispensing of other psychotropic medications (antidepressants, Stimulants) without prescriptions to avoid any reporting, since all these medications are also available over the counter, hence we might have under-reported the prescribing trends of other psychotropic medications at our local pharmacies.

## 6 Conclusions

Dispensing of psychotropic medications without a prescription by retail pharmacies is a common practice in Karachi. Benzodiazepines, by far, were the most common dispensed and recommended psychotropic medications, with most of the dispensers not being aware of the abuse potential of these control medications. The importance of qualification, knowledge, and training of pharmacy employees is not highlighted in Pakistan. Effective implementation of policies is needed. Medication sales should be monitored, and their availability should be limited in a way that can benefit the population and reduce harm.

## Declarations

### Author contribution statement

Shahzain Hasan: Conceived and designed the experiments; Performed the experiments; Analyzed and interpreted the data; Contributed reagents, materials, analysis tools or data; Wrote the paper.

Mujeeb U. Shad: Conceived and designed the experiments; Contributed reagents, materials, analysis tools or data; Wrote the paper.

### Funding statement

This research did not receive any specific grant from funding agencies in the public, commercial, or not-for-profit sectors.

### Data availability statement

Data will be made available on request.

### Declaration of interest’s statement

The authors declare no conflict of interest.

### Additional information

Supplementary content related to this article has been published online at [URL].

## Appendix A Supplementary data

Questionnaire Questionnaire
