# Supplementary material for: Dispensing practices for psychotropic medications amongst pharmacists in Karachi, Pakistan
Source: Heliyon. 2022 Oct 28;8(11):e11298. doi: 10.1016/j.heliyon.2022.e11298 (PMC9636556; doi:10.1016/j.heliyon.2022.e11298)
Supplement: Questionnaire [file mmc1.docx]

**Dispensing Practices for Psychotropic Medications Amongst Pharmacists in Karachi, Pakistan**.

**Questionnaire:**

1) Age of the Participant:

2) Gender

1= Male, 2= Female

3) Location

1= Hospital Pharmacy, 2= Clinic Pharmacy, 3= Independent retail Pharmacy

4) Level of Education

1= Matriculation, 2= Intermediate level, 3= Higher Education Other than Bachelors,

4= Bachelors or higher

5) Higher Education degree

1= Doctor of Pharmacy, 2= Other degree in pharmacy, 3= Degree other than pharmacy, 4= None

6) Number of years worked at the pharmacy

1= <5 years, 2= 5-10 years, 3= >10 years

7) How often do the patients ask you for recommending medications

1= Every day, 2= Rarely, 3= Never

8) What is the most common medication that you have dispensed for behavioral and psychiatric issues?

1- Benzodiazepine/Anxiolytics, 2= Anti-depressants, 3=Antipsychotics, 4= Stimulants

9) How often do you recommend a medication for behavioral issues?

1- Every day, 2= Rarely, 3= Never

10) What is the most common medication that you have recommended for behavioral and psychiatric issues?

1= Benzodiazepine/Anxiolytics, 2=Anti-depressants, 3=Antipsychotics, 4=Stimulants, 5= None

11)Reason for recommending psychotropic medications

1= Self-prescribed a new medication for a diagnosed psychiatric condition,

2= Self-prescribed a new medication for a patient without diagnosis.

3= Never Recommend

12) Do you dispense medications according to prescriptions?

1= Always, 2= Sometimes, 3= No prescription needed

13) What is the most common psychiatric condition for which you have dispensed a medication?

1= Anxiety, 2= depression, 3= Psychosis

14)What describes your knowledge of the condition for which the drug was prescribed?

1= No knowledge,

2= Know a little about the disorder and the commonly prescribed medications

3= Sure about the diagnosis and treatment

15) Why Do you think dispensing psychotropic medications might help the patient without a prescription?

1= Increased Burden of mental issues

2= Overwhelming number of cases of the disorders

3= Patients can avoid long waiting’s in the clinics and can get their problems sorted out easily

4= Never Dispense without Prescription

16) Are you aware of the abuse potential of these medications?

1= Yes, I am aware of that

2= No, I am not aware of that

3= These medications do not have abuse potential.

**صنف**

**1 = مرد ، 2 = عورت۔**

2**) عمر**

**3) مقام۔**

1 = ہسپتال فارمیسی ، 2 = کلینک فارمیسی ، 3 = آزاد خوردہ فارمیسی۔

4**) تعلیم کی سطح**

1 = میٹرک ،

2 = انٹرمیڈیٹ لیول ،

3 = بیچلرز کے علاوہ اعلیٰ تعلیم ،

4 = بیچلرز یا اس سے زیادہ۔

5**) بیچلر کی ڈگری**

1 = ڈاکٹر آف فارمیسی ،

2 = فارمیسی میں دوسری ڈگری ،

3 = فارمیسی کے علاوہ ڈگری ،

4 = کوئی نہیں

6**) فارمیسی میں کام کرنے والے سالوں کی تعداد۔**

1 = <5 سال ، 2 = 5-10 سال ، 3 => 10 سال۔

7**) مریض آپ سے کتنی بار ادویات تجویز کرنے کے لیے کہتے ہیں؟**

1 = ہر روز ،

2 = شاذ و نادر ،

3 = کبھی نہیں۔

8**) سب سے عام دوا کون سی ہے جو آپ نے رویے اور نفسیاتی مسائل کے لیے دی ہے؟**

1- Benzodiazepine/Anxiolytics، 2 = Anti-depressants، 3 = Antipsychotics، 4 = stimulants

9) **آپ رویے کے مسائل کے لیے کتنی بار دوا کی سفارش کرتے ہیں؟**

1- ہر روز ، 2 = شاذ و نادر ، 3 = کبھی نہیں۔

10**) سب سے عام دوا کون سی ہے جو آپ نے رویے اور نفسیاتی مسائل کے لیے تجویز کی ہے؟**

1 = Benzodiazepine/Anxiolytics، 2 = Anti-depressants، 3 = Antipsychotics، 4 = stimulants، 5 = کوئی نہیں

11**) نفسیاتی ادویات تجویز کرنے کی وجہ۔**

1 = نفسیاتی تشخیص شدہ نفسیاتی حالت کے لیے ایک نئی دوا ،

2 = بغیر تشخیص کے مریض کے لیے نئی دوا خود تجویز کی۔

3 = کبھی سفارش نہ کریں۔

**12) کیا آپ نسخے کے مطابق ادویات دیتے ہیں؟**

1 = ہمیشہ ، 2 = کبھی کبھی ، 3 = کسی نسخے کی ضرورت نہیں۔

13**) سب سے عام نفسیاتی حالت کیا ہے جس کے لیے آپ نے دوا دی ہے؟**

1 = بے چینی ، 2 = افسردگی ، 3 = نفسیات۔

14**) اس حالت کے بارے میں آپ کا علم کیا بیان کرتا ہے جس کے لیے دوا تجویز کی گئی تھی؟**

1 = علم نہیں ،

2 = خرابی کی شکایت اور عام طور پر تجویز کردہ ادویات کے بارے میں تھوڑا جانیں۔

3 = تشخیص اور علاج کے بارے میں یقین ہے۔

**15) آپ کے خیال میں نفسیاتی ادویات کی ترسیل مریض کی نسخے کے بغیر کیوں مدد کر سکتی ہے؟**

1 = ذہنی مسائل کا بڑھتا ہوا بوجھ۔

2 = خرابیوں کے معاملات کی زبردست تعداد۔

3 = مریض کلینک میں طویل انتظار سے بچ سکتے ہیں اور اپنے مسائل کو آسانی سے حل کر سکتے ہیں۔

**16) کیا آپ ان ادویات کے غلط استعمال سے واقف ہیں؟**

1 = ہاں ، میں اس سے واقف ہوں۔

2 = نہیں ، میں اس سے واقف نہیں ہوں۔

3 = ان ادویات میں غلط استعمال کی صلاحیت نہیں ہے۔
